# Correlates of In-Hospital Deaths among Hospitalizations with Pulmonary Embolism: Findings from the 2001−2008 National Hospital Discharge Survey

**DOI:** 10.1371/journal.pone.0034048

**Published:** 2012-07-06

**Authors:** James Tsai, Scott D. Grosse, Althea M. Grant, Nimia L. Reyes, W. Craig Hooper, Hani K. Atrash

**Affiliations:** Division of Blood Disorders, National Center on Birth Defects and Developmental Disabilities Centers for Disease Control and Prevention, Atlanta, Georgia, United States of America; Leiden University Medical Center, The Netherlands

## Abstract

**Background:**

Deep vein thrombosis and pulmonary embolism (PE) are responsible for substantial mortality, morbidity, and impaired health-related quality of life. The aim of this study was to evaluate the correlates of in-hospital deaths among hospitalizations with a diagnosis of PE in the United States.

**Methods:**

By using data from the 2001−2008 National Hospital Discharge Survey, we assessed the correlates of in-hospital deaths among 14,721 hospitalizations with a diagnosis of PE and among subgroups stratified by age, sex, race, days of hospital stay, type of admission, cancer, pneumonia, and fractures. We produced adjusted rate ratios (aRR) and 95% confidence intervals using log-linear multivariate regression models.

**Results:**

Regardless of the listing position of diagnostic codes, we observed an increased likelihood of in-hospital death in subgroups of hospitalizations with ages 50 years and older (aRR = 1.82−8.48), less than 7 days of hospital stay (aRR = 1.43−1.57), cancer (aRR = 2.10−2.28), pneumonia (aRR = 1.79−2.20), or fractures (aRR = 2.18) (except for first-listed PE), when compared to the reference groups with ages 1−49 years, 7 days or more of hospital stay, without cancer, pneumonia, or fractures while adjusting for covariates. In addition, we observed an increased likelihood of in-hospital death for first-listed PE in hospitalizations of women, when compared to those of men (aRR = 1.45).

**Conclusions:**

The results of this study provide support for identifying, developing, and implementing effective, evidence-based clinical assessment and management strategies to reduce PE-related morbidity and mortality among hospitalized PE patients who may have concurrent health conditions including cancer, pneumonia, and fractures.

## Introduction

Deep vein thrombosis (DVT) and pulmonary embolism (PE) are part of a venous thromboembolism spectrum with a complex and multifaceted etiology involving the interactions of biological, behavioral, health, and environmental risk factors [1]–[4]. The *2008 Surgeon General’s Call to Action to Prevent Deep Vein Thrombosis and Pulmonary Embolism* suggests that DVT and PE may contribute to as many as 100,000−180,000 deaths per year [5]. Furthermore, PE can result in substantial morbidities including recurrence, chronic pulmonary hypertension, disability, and impaired health-related quality of life [5]–[8]. Accumulating evidence suggests that hospitalized patients including those with cancer, pneumonia, and fractures may have a high risk of PE [1], [9]–[11]. These health conditions were found among the leading diagnostic categories and linked to approximately 3.3 million hospitalizations in the United States in 2007 [12]. Recent data from the National Hospital Discharge Survey (NHDS) showed that the overall case-fatality rate of hospitalizations with a PE diagnosis declined from 11.4% to 7.1% during 2001−2008, even though a corresponding reduction in the estimated number of in-hospital deaths among such hospitalizations was not observed for the same period [13].

To date, numerous clinical and epidemiologic studies have assessed PE mortality by using data ascertained from death certificates, autopsy records, or participants of clinical investigations [14]–[17]. A number of studies have assessed the case-fatality rates of PE and the relationship between in-hospital death and risk factors among patients or hospitalizations with a PE diagnosis by using the national hospital discharge surveys [13], [18]–[24]. However, few studies have examined the correlates of in-hospital death, encompassing diagnostic categories of cancer, pneumonia, and fractures, among a nationally representative sample of hospitalizations with a PE diagnosis in the United States. Such a study is valuable to identify risk factors for PE-related death and to provide observational evidence that can inform and improve clinical assessment and intervention strategies for hospitalized PE patients. Therefore, the aim of this study was to assess the correlates of in-hospital death, including demographic characteristics and clinical risk factors, among hospitalizations with a PE diagnosis (i.e., first-listed and any-listed) in the United States by analyzing data from the 2001−2008 NHDS [25].

## Methods

### Data Source

The NHDS was conducted annually from 1965–2010 by the National Center for Health Statistics (NCHS), Centers for Disease Control and Prevention (CDC) to collect demographic and medical information of a sample of hospitalizations from a national probability selection of hospitals in the 50 states and the District of Columbia [26]. The NHDS was designed to collect information on characteristics of inpatients discharged from non-Federal short-stay hospitals in the United States. The NHDS includes general or children’s general hospitals with an average length of stay of fewer than 30 days for all patients. Federal, military, and Department of Veterans Affairs hospitals were excluded, as were hospital units of institutions and hospitals with fewer than 6 beds staffed for patient use. Details about the survey methodology are available elsewhere [25]. The NHDS collects information of hospitalizations including age, race, sex, length of hospital stay, type of admission (e.g., emergency, urgent, and elective), discharge status (e.g., death), medical diagnosis and procedure codes. Data collection for the NHDS was approved by the NCHS Research Ethics Review Board (NCHS ERB #2011–12). Analysis of deidentified data from the survey is exempt from the federal regulations for the protection of human research participants.

### Hospitalization Records

Because persons with multiple hospitalizations during the survey year may be sampled more than once, estimates from the NHDS are for hospitalizations, not for persons. Each sampled hospitalization has a maximum of seven diagnostic codes based on the International Classification of Diseases, Ninth Revision, Clinical Modification (ICD-9-CM). The first-listed code is a principal diagnosis. We limited our analysis to a sample of non-newborn hospitalizations with a first-listed PE (n = 8,990) or any-listed PE (n = 14,721). Table 1 lists the codes for identification of PE and selected diagnostic categories for the years 2001−2008. In addition to demographic variables (i.e., age, sex, and race), we also included covariates such as cancer, pneumonia, and fractures in the analysis. We included these covariates because they are well-established independent risk factors for PE and may represent important pathways for prevention.

**Table 1:** The International Classification of Diseases, Ninth Revision, Clinical Modification (ICD-9-CM) codes for identification of selected medical diagnoses, 2001−2008.

**Description**	**ICD-9-CM codes** a
**Pulmonary embolism**	415.1×, 634.6×, 635.6×, 636.6×, 637.6×, 638.6, and 673.2×
**Cancer** (malignant neoplasm)	140–208, 230–234
**Pneumonia**	480–486
**Fractures**	800–829

a Only three-digit category codes are listed for cancer, pneumonia, and fractures.

### Statistical Analysis

We combined 8 years of data to increase statistical reliability and to allow the analysis of some subpopulation groups that otherwise would have been too small to produce statistically reliable estimates [25], [27]. We estimated the case-fatality rates in hospitalizations with a diagnosis of PE (i.e., first-listed and any-listed) and in subgroups stratified by age, sex, race, days of hospital stay, type of admission, and status of cancer, pneumonia, and fractures [28]. To examine the associations between in-hospital death and risk factors among hospitalizations with a PE diagnosis during the period 2001−2008, we produced unadjusted and adjusted rate ratios (aRRs) with 95% confidence intervals (CIs) by using demographic and medical risk factors as predictors; status of in-hospital death was used as an outcome variable in multivariate log-linear regression models.

To present all estimates that fully account for multiple stages of sampling, stratification, and clustering design of the NHDS, we accessed the restricted datasets through the Research Data Center (CDC, Atlanta, GA, 2011) [26]. We used SPSS 19 Complex Samples for Survey Analysis (IBM Corp., Armonk, NY, 2010) and STATA 11 (StataCorp LP, College Station, TX, 2009) to perform the data management and analyses to account for complex sample survey design [29].

## Results

Except for a limited number of deaths in hospitalizations of fractures with first-listed PE, the case-fatality rates varied significantly by age, status of cancer, pneumonia, and fractures among hospitalizations with first-listed PE and any-listed PE (P<0.05 for Wald-F test). Specifically, the case-fatality rates were higher among hospitalizations with advanced ages, cancer, pneumonia, or fractures than among those with younger ages, without a diagnosis of cancer, pneumonia, or fractures (P<0.05 for Wald-F test) (Table 2). Of those hospitalizations with first-listed PE, we observed increased likelihoods of in-hospital death in subgroups with ages 50−79 years (aRR = 3.63; CI: 1.88−7.02), ages 80 years and older (aRR = 8.48; CI: 4.03−17.90), female (aRR = 1.45; CI: 1.09−1.93), less than 7 days of hospital stay (aRR = 1.57; CI: 1.12−2.21), cancer (aRR = 2.28; CI: 1.68−3.09), or pneumonia (aRR = 2.20; CI: 1.28−3.77), when compared to the reference groups with ages 1−49 years, male, 7 days or more of hospital stay, without cancer or pneumonia while adjusting for all covariates (Table 2). Similarly, of hospitalizations with any-listed PE diagnosis, we observed increased likelihoods of in-hospital death among subgroups with ages 50–79 years (aRR = 1.82; CI: 1.47−2.25), ages 80 years and older (aRR = 3.26; CI: 2.58−4.12), race/ethnicity listed under “other” (aRR = 1.36; CI: 1.15−1.76), less than 7 days of hospital stay (aRR = 1.43; CI: 1.15−1.76), cancer (aRR = 2.10; CI: 1.74−2.53), pneumonia (aRR = 1.79; CI: 1.48−2.17), or fractures (aRR = 2.32; CI: 1.68−3.20), when compared to the reference groups with ages 1−49 years, male, white, 7 days or more of hospital stay, without cancer, pneumonia, or fractures after adjustment of all covariates (Table 2).

**Table 2:** Estimated case-fatality rates and rate ratios for in-hospital death among hospitalizations with a PE diagnosis, 2001−2008, NHDS, United States.

Characteristics		In-hospital death							
		First-listed PE (n = 8,990)				Any-listed PE (n = 14,721)			
	n^a^	Case-fatality rate (%)(95% CI)^b^	*P*-value^c^	UnadjustedRR^d^ (95% CI)	AdjustedRR^e^ (95% CI)	Case-fatalityrate (%)(95% CI)	*P*-value	UnadjustedRR (95% CI)	AdjustedRR (95% CI)
**Age**			*P*<0.001				*P*<0.001		
1−49	3,550	1.0 (0.5−1.9)		Referent^f^	Referent	4.0 (3.3−4.8)		Referent	Referent
50−79	8,240	3.8 (3.2−4.5)		3.82 (2.01−7.31)	3.63 (1.88−7.02)	8.1 (7.1−9.1)		2.02 (1.64−2.49)	1.82 (1.47−2.25)
≥80	2,931	8.2 (5.8−11.3)		8.23 (4.03−16.8)	8.48 (4.03−17.9)	13.6 (11.6−16.0)		3.41 (2.67−4.35)	3.26 (2.58−4.12)
**Sex**			*P* = 0.015				*P* = 0.884		
Male	6,224	3.1 (2.5−3.7)		Referent	Referent	8.3 (7.9−9.2)		Referent	Referent
Female	8,497	4.5 (3.5−5.6)		1.46 (1.08−1.96)	1.45 (1.09−1.93)	8.2 (7.2−9.4)		0.99 (0.86−1.14)	1.00 (0.86−1.15)
**Race**			−				−g		
White	7,933	4.0 (3.2−4.9)		Referent	Referent	8.5 (7.4−9.9)		Referent	Referent
Black	2,097	3.6 (2.9−4.6)		0.92 (0.69−1.22)	1.17 (0.88−1.57)	7.4 (6.6−8.4)		0.87 (0.72−1.05)	1.10 (0.91−1.32)
Other	395	−h		−	−	10.6 (9.2−12.2)		1.24 (1.01−1.54)	1.36 (1.14−1.64)^f^
Not stated	4,296	3.7 (2.4−5.6)		0.93 (0.57−1.51)	0.96 (0.59−1.55)	7.7 (6.5−9.0)		0.90 (0.72−1.12)	0.93 (0.75−1.17)
**Days of hospital stay**			*P* = 0.153				*P* = 0.168		
≥7 days	6,746	3.4 (2.6−4.3)		Referent	Referent	7.6 (6.5−8.8)		Referent	Referent
<7 days	7,975	4.2 (3.4−5.1)		1.24 (0.92−1.67)	1.57 (1.12−2.21)	8.8 (7.7−10.0)		1.15 (0.94−1.41)	1.43 (1.15−1.76)
**Type of Admission**			*P* = 0.135				*P* = 0.228		
Emergency	9,120	4.1 (3.4−5.1)		Referent	Referent	8.4 (7.5−9.4)		Referent	Referent
Urgent	2,545	2.9 (2.1−4.1)		0.71 (0.50−1.02)	0.67 (0.47−0.96)	7.4 (6.0−9.1)		0.88 (0.70−1.11)	0.84 (0.67−1.05)
Elective	1,643	2.9 (1.1−7.3)		0.69 (0.26−1.85)	0.63 (0.24−1.61)	7.6 (5.2−10.9)		0.90 (0.62−1.31)	0.93 (0.65−1.34)
Not stated	1,413	5.4 (3.7−7.7)		1.31 (0.86−1.98)	1.33 (0.88−2.02)	10.0 (8.1−12.3)		1.19 (0.94−1.51)	1.23 (0.96−1.56)
**Cancer**			*P*<0.001				*P*<0.001		
No	12,233	3.3 (2.7−4.2)		Referent	Referent	7.1 (6.3−8.1)		Referent	Referent
Yes	2,488	7.5 (6.1−9.2)		2.25 (1.62−3.13)	2.28 (1.68−3.09)	14.1 (12.5−15.8)		1.97 (1.64−2.37)	2.10 (1.74−2.53)
**Pneumonia**			*P* = 0.012				*P*<0.001		
No	12,898	3.6 (3.0−4.3)		Referent	Referent	7.5 (6.6−8.4)		Referent	Referent
Yes	1,823	7.1 (4.3−11.6)		2.00 (1.17−3.43)	2.20 (1.28−3.77)	13.7 (11.5−16.3)		1.83 (1.50−2.24)	1.79 (1.48−2.17)
**Fractures**			−				*P*<0.001		
No	14,354	3.9 (3.3−4.7)		−	−	7.9 (7.2−8.8)		Referent	Referent
Yes	367	−		−	−	17.8 (13.1−23.7)		2.24 (1.67−3.00)	2.32 (1.68−3.20)

a Maximum subgroup sample size.

b Confidence interval.

c *P*-value for Wald-F test.

d Rate ratio.

e Rate ratio from log-linear regression model that adjusted for age, sex, race, days of stay, type of admission, cancer, pneumonia, and fractures.

f Referent rate ratio = 1.00.

g Estimate may not be reliable due to underreporting of race information.

h Unstable estimate due to small subgroup size.

## Discussion

Previous studies have assessed many correlates of in-hospital death among PE patients in healthcare settings [21]–[24], our study expanded previous research by assessing in-hospital deaths with a PE diagnosis encompassing first-listed and any-listed ICD-9 diagnostic codes during 2001−2008. The associations of cancer, pneumonia, and fractures with in-hospital deaths among a nationally representative sample of hospitalizations with a first-listed and any-listed PE diagnosis in the United States during the study period have not been reported in the past. Such observational evidence is valuable for identifying risk factors that may place patients at an increased risk of death and for improving clinical assessment and management to prevent fatal PE. Regardless of the listing position, we observed an increased likelihood of in-hospital death in subgroups of hospitalizations with advanced ages, less than 7 days of hospital stay, cancer, pneumonia, or fractures (except for first-listed PE), when compared to the reference groups with ages 1−49 years, 7 days or more of hospital stay, without cancer, pneumonia, or fractures while adjusting for all covariates. In addition, we observed an increased likelihood of in-hospital death for first-listed PE in hospitalizations of women when compared to those of men. However, the exact reason for this increase remained unknown, additional research may be needed. Interestingly, regardless of the listing position, we did not find that type of admission (e.g., emergency admission) was a factor independently associated with in-hospital death with a PE diagnosis.

Some results of this study were consistent with evidence that mortality rates were high among patients with advanced ages or co-morbid conditions such as cancer. Recent data suggested that a decline in the overall case-fatality rate of US hospitalizations with a PE diagnosis could be attributable to a combination of an increased number of PE diagnoses resulting from the widely used computed tomography pulmonary angiography (e.g., detection of asymptomatic PE or small peripheral emboli) together with more effective treatment and fewer complications [13], [20], [30]–[32]. Regardless, the strength of associations between the correlates and in-hospital death remained essentially unchanged during the study period, when survey year was used as an additional stratification variable or as a covariate in the regression models (data not shown).

The results of our study provide support for enhancing efforts toward identifying and implementing appropriate preventive strategies among hospitalized patients, including those with co-morbid conditions such as cancer, pneumonia, and fractures in healthcare settings [33]. Because of the disease severity, fatal PE may occur early during a hospital stay and may be undiagnosed, clinicians must be attentive to identify PE patients. Because the case-fatality rate was higher among hospitalizations of a shorter hospital stay than those of a longer stay, our results suggest that fatal PE may occur early after a hospital admission [34], [35]. As such, clinical assessment, diagnostic evaluation, and appropriate treatment for PE should be performed early and promptly among hospitalized patients [34], [36], [37].

Our study has some limitations. Cross-sectional surveys are not designed to evaluate a cause-effect relationship. The NHDS hospitalizations rates do not necessarily reflect rates per person, as hospitalizations of recurrent PE patients could be included. Because asymptomatic PE patients with other co-morbid conditions might be undiagnosed or misdiagnosed, or PE diagnosis might have not been made before death occurred, hospitalizations for these patients might not have been included in our study. Due to underreporting of race in the NHDS, extra caution is needed for making any statistical inference regarding race as such estimates may not be reliable [28]. Even though we adjusted for many covariates in the study, we were unable to control for potential anticoagulant therapies, medications, and other co-morbidities that may affect short-term mortality in PE patients, due to the constraints of administrative datasets.

The results from this analysis and earlier study suggest that a majority of in-hospital deaths with a PE diagnosis could be attributable to concurrent PE and other health conditions [13]. Presently, there is a paucity of information concerning the clustering of risk factors that may hinder timely diagnosis, obscure clinical assessment and management, and exacerbate the risk of morbidity, mortality, and impaired health-related quality of life in PE patients [38]–[42]. Given that PE is a continuing clinical and public health concern in the United States, additional research is needed to elucidate amenable or manageable risk factors that are associated with devastating health consequences including death among PE patients. As the US population is aging, the co-existence of two or more chronic health conditions is a growing public health problem and poses enormous challenges for clinicians, patients, and healthcare systems [43]–[45]. To effectively reduce PE-related mortality and to improve overall health and well-being among most PE patients, more epidemiologic research and multimorbidity intervention studies are needed in order to deliberate, investigate, inform, and advance evidence-based practice in healthcare settings where integrated interventions may be coordinated and delivered to patients with multiple health conditions including PE [46]–[48].

In conclusion, the results of this study provide support for identifying, developing, and implementing effective, evidence-based clinical assessment and management strategies to reduce PE-related morbidity and mortality among hospitalized PE patients who may have concurrent health conditions including cancer, pneumonia, and fractures.
